# Anti-inflammatory effects of new human histamine H_3_ receptor ligands with flavonoid structure on BV-2 neuroinflammation

**DOI:** 10.1007/s00011-022-01658-z

**Published:** 2022-11-12

**Authors:** Ewelina Honkisz-Orzechowska, Katarzyna Popiołek-Barczyk, Zuzanna Linart, Jadwiga Filipek-Gorzała, Anna Rudnicka, Agata Siwek, Tobias Werner, Holger Stark, Jakub Chwastek, Katarzyna Starowicz, Katarzyna Kieć-Kononowicz, Dorota Łażewska

**Affiliations:** 1 Faculty of Pharmacy, Department of Technology and Biotechnology of Drugs, Jagiellonian University Medical College in Kraków, Medyczna 9, 30-688 Kraków, Poland; 2 Department of Neurochemistry, Maj Institute of Pharmacology, Polish Academy of Sciences, Smętna 12, 31-343 Kraków, Poland; 3 Faculty of Pharmacy, Department of Pharmacobiology, Jagiellonian University Medical College in Kraków, Medyczna 9, 30-688 Kraków, Poland; 4 Institute of Pharmaceutical and Medicinal Chemistry, Heinrich Heine University Duesseldorf, Universitaetsstr. 1, 40225 Duesseldorf, Germany

**Keywords:** Microglia, Neuroinflammation, Histamine H_3_ receptor, Chalcone

## Abstract

**Objective:**

Microglia play an important role in the neuroinflammation developed in response to various pathologies. In this study, we examined the anti-inflammatory effect of the new human histamine H_3_ receptor (H_3_R) ligands with flavonoid structure in murine microglial BV-2 cells.

**Material and methods:**

The affinity of flavonoids (**E243** -flavone and **IIIa**–**IIIc**—chalcones) for human H_3_R was evaluated in the radioligand binding assay. The cytotoxicity on BV-2 cell viability was investigated with the MTS assay. Preliminary evaluation of anti-inflammatory properties was screened by the Griess assay in an in vitro neuroinflammation model of LPS-treated BV-2 cells. The expression and secretion of pro-inflammatory cytokines were evaluated by real-time qPCR and ELISA, respectively. The expression of microglial cell markers were determined by immunocytochemistry.

**Results:**

Chalcone derivatives showed high affinity at human H_3_R with *K*_*i*_ values < 25 nM. At the highest nontoxic concentration (6.25 μM) compound **IIIc** was the most active in reducing the level of nitrite in Griess assay. Additionally, **IIIc** treatment attenuated inflammatory process in murine microglia cells by down-regulating pro-inflammatory cytokines (IL-1β, IL-6, TNF-α) at both the level of mRNA and protein level. Our immunocytochemistry studies revealed expression of microglial markers (Iba1, CD68, CD206) in BV-2 cell line.

**Conclusions:**

These results emphasize the importance of further research to accurately identify the anti-inflammatory mechanism of action of chalcones.

## Introduction

Microglia are called the ‘third element’ of the central nervous system (CNS), along with neurons and astrocytes, since in 1918 Pio del Rio Hortega developed a method to stain microglia and distinguish them from the neighbouring cells of the CNS [1]. As the first and main form of active innate immune defence in the CNS, this unique population of macrophage-like cells is responsible for homeostasis in the brain microenvironment, responds to an external signal that essentially determines their activities [2]. Recently, the specific role of microglia in neuroinflammation has been discussed [3]. Exposure of the microglia to infections, injuries, or endogenously released neurotoxic factors triggers the microglia to undergo a directed migration towards dead or dying neurons [4]. This process is called microglia activation and is associated with the production and secretion of a variety of mediators such as cytokines, reactive oxygen species (ROS), reactive nitrogen species (RNS), matrix metalloproteinases, and prostaglandins. Microglial heterogeneity within the CNS is particularly visible in their morphology and expression of cellular markers, which determines the role of those cells in physiological or pathological conditions. The most widely used markers of microglial cells are ionized calcium-binding adapter molecule 1 (Iba1) and cluster of differentiation receptors (e.g. CD68, CD206). Activated microglia are desirable in the regulation of tissue repair and recovery. However, excessive or chronic activation can lead to harmful effects [5]. The contribution of microglia to inflammatory changes in the brains of patients with Alzheimer’s disease (AD) has been widely discussed in the last decade. AD is a devastating neurodegenerative disease that is associated with loss of cognitive functioning and behavioural ability. Very recently, Pascoal et al. using positron emission tomography imaging demonstrated in living patients with Alzheimer’s disease that activation of microglial cells in the brain is not only a result of disease progression, but is a key upstream mechanism required for disease development [6]. This research supports many of the earlier studies that pointed to an important role for microglia in neuroinflammation [7, 8].

Histamine is an important neurotransmitter and exerts its action through four receptors: histamine H_1_ receptors (H_1_R), histamine H_2_ receptors (H_2_R), histamine H_3_ receptors (H_3_R), and histamine H_4_ receptors (H_4_R). Among them, H_1_R, H_2_R, and H_3_R are observed in the brain, whereas the results about H_4_R are inconsistent [9]. H_3_R due to its distribution in the CNS is an interesting target for the search for new therapy for CNS disorders. Numerous pharmacological studies show that blocking this receptor provides beneficial effects in the treatment of neurodegenerative diseases (e.g., AD, Parkinson's disease, PD; Huntington’s disease), attention deficit hyperactivity disorder (ADHD), neuropathic pain, or multiple sclerosis (MS) (review by [10, 11]). Originally, Arrang et al*.* showed that H_3_R is a presynaptic autoreceptor present in histaminergic neurons of the CNS as it mediates histamine inhibition by its release [12]. Furthermore, H_3_R also occurs in non-histaminergic neurons of the CNS and the autonomic nervous system and acts as heteroreceptors that regulate the release of other neurotransmitters such as dopamine, serotonin, norepinephrine, acetylcholine, γ-amino butyric acid, and glutamate [13, 14]. According to this evidence, modulation of the histaminergic system in the brain could play an important role in the pathophysiology of brain disorders. Furthermore, the neuroprotective activity of histamine receptor ligands can increase pharmacological effectiveness by preventing loss of neurons and slowing disease progression (review by [15]). This therapeutic activity could be the result of histaminergic neurotransmission. Neurotransmitters are released through a presynaptic inhibitory mechanism. Several studies indicated that by blocking H_3_R and thus the feedback loop mechanism, the release of neurotransmitters can be increased [14, 16].

Many preclinical studies have shown the positive impact of H_3_R antagonists/inverse agonists on memory impairments in various models, both in vitro and in vivo studies. Ciproxifan, an imidazole-based H_3_R reference antagonist, demonstrated improvements in hyperactivity and memory deficits after administration of this drug in a mouse model of AD [17]. The other compound JNJ-10181457 (inverse agonist of H_3_R) reversed memory impairments caused by scopolamine in rats [18]. Further ex vivo and in vivo studies with this compound by Iida et al*.* displayed inhibition of microglia activation and reduction of increased levels of inflammatory cytokines and depression-like behaviour in mice caused by lipopolysaccharide (LPS) [19]. Several studies also showed the positive effects of H_3_R antagonists/inverse agonists not only in improving cognition impairments, but also in protecting against neuronal loss. In APP/PS1 mice model of AD, thioperamide (H_3_R antagonist/H_4_R antagonist) activated a cyclic AMP-response element-binding protein (CREB) and thus mediated autophagy and lysosomal pathway [20]. Further studies by this research group, in this mice model of AD, demonstrated inhibition of gliosis and inflammation by thioperamide through activation the cAMP/PKA/CREB pathway [21]. Another compound, clobenpropit (H_3_R antagonist/H_4_R partial agonist), was evaluated in the ICR mice model with memory deficit-induced LPS. Pretreatment for 30 days with clobenpropit showed neuroprotection of this compound against neuronal inflammation and mitochondrial impairment [22]. Furthermore, in human post mortem cases of PD, increased histaminergic nerve fibers associated with up-regulation of H_3_R were observed [23]. The same study also showed that chronic administration of H_3_R ligands pitolisant (BF2.649, Wakix®; H_3_R inverse agonist) and clobenpropit, significantly reduced brain pathology by lowering α-synuclein and phosphorylated τ-protein in cerebrospinal fluid. Some of the most promising H_3_R ligands such as, e.g., GSK239512, MK-0249, ABT-288, SAR110894 or pitolisant entered clinical trials for the potential treatment of AD, dementia, ADHD, narcolepsy, multiple sclerosis, obesity, or schizophrenia [10, 11]. The published results of some of these studies showed a tolerable safety profile of compounds and improvements in some domain of cognition (e.g. episodic memory in patients with mild to moderate AD: GSK239512 [24, 25] or no cognitive enhancing effects (e.g. MK-0249 [26], ABT-288 [27]). The satisfactory results of pitolisant in the treatment of narcoleptic patients allowed the European Medicines Agency (EMA) (2016) and the US Food and Drug Administration (2019) to approve it as the drug Wakix® [28] and recently also for the treatment of excessive daytime sleepiness as the drug Ozawade by EMA (2021) [29]. Promising results in promoting wakefulness and cognition enhancement led to the study of potential therapeutic benefits of pitolisant in other CNS disorders. Trenite et al*.* studied the effect of pitolisant in patients with epilepsy and showed a significant suppressive effect in epileptic discharges [30]. In agreement are also the study by Sadek et al*.* that showed that several synthetic H_3_R antagonists showed anticonvulsant effects against maximum electroshock-induced and pentylenetetrazole-induced convulsions in rats that have phenytoin as the reference anti-epileptic drug [31].

Flavonoids (flavones, chalcones) are a promising group of compounds with wide biological activities [32]. Especially interested are their abilities related to the treatment of neurodegenerative disorders. Some flavonoids tested in in vivo AD models (e.g., quercetin, apigenin) showed very favourable activity [33]. These effects could be associated with antioxidant, anti-inflammatory, or anti-apoptotic properties [32]. Chalcones (aromatic α,ß-unsaturated ketones) are open chain bioprecursors of flavonoids (e.g., flavones). Chalcones also have a broad range of biological activities [34]. In general, their activity is related to the inhibitory effect on COX-1, COX-2, β-glucuronidase. They showed anti-inflammatory activity by suppressing NO and PGE-2 production, a commonly assessed neuroinflammatory endpoint and a common mediator of brain inflammation, respectively, in LPS-stimulated microglia.

This work explored the hypothesis that H_3_R ligands with the flavonoids’ scaffold may suppress inflammation induced by LPS activated microglia cells. The expression of H_3_R in primary (mouse/rat) microglia was confirmed previously by several research teams [19, 35, 36]. In our work, we investigated the potential of H_3_R ligands to control microglial functions for therapeutic purposes. For studies, we chosen methoxychalcone derivatives and a flavone compound.

## Material and methods

### Synthesis of tested compounds E243 and IIIa–IIIc

**Fig. 1:**
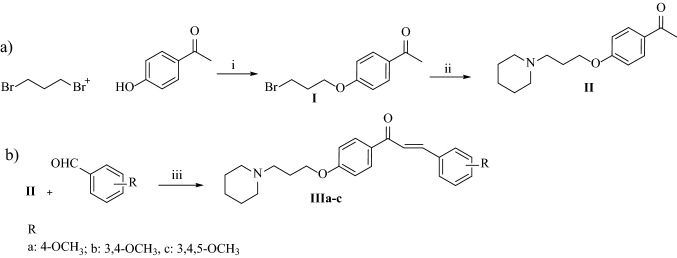
Synthetic route of compounds **IIIa-IIIc**. (i) K_2_CO_3_, acetone, 48 h; reflux; (ii) piperidine, K_2_CO_3_, KI, CH_3_CN, 24 h, reflux; (iii) 15% KOH, EtOH, 0.5–4 h, rt

Compound **E243** was synthesized as described by Bajda et al*.* [37]. Compounds **IIIa**–**IIIc** were obtained (as seen in Fig. 1) by the Claisen-Schmidt condensation of 1-(4-(3-piperidin-1-yl)propoxy)phenyl)ethan-1-one (**II**) with an appropriate aldehyde in the presence of 15% KOH in absolute ethanol. 1-(4-(3-Piperidin-1-yl)propoxy)phenyl)ethan-1-one (**II**) was prepared from 1-(4-(3-bromopropoxy)phenyl)ethan-1-one (**I**) in a nucleophilic substitution of a piperidine with good yield (59%). The reaction was conducted in the presence of potassium carbonate in acetonitrile. Starting bromide **I** was prepared by *O*-alkylation of commercially available 4-hydroxyacetophenone with 1,3-dibromopropane refluxed in acetone with the powdered potassium carbonate. The final compounds were obtained as salts of hydrochloric acid. Their purity and identity were confirmed by spectral and chromatographic analysis (^1^H NMR, ^13^C NMR, LC–MS).

### Synthesis of 1-(4-(3-bromopropoxy)phenyl)ethan-1-one (I)

4-Hydroxyacetophenone (0.1 mol; 13.62 g) was added to 100 ml of acetone, followed by potassium carbonate (0.16 mol; 22.11 g) and refluxed for 10 min. Next, 1,3-dibromopropane was slowly added and the solution was further refluxed for 7 h. Then, the solid was filtered and the filtrate evaporated. 100 ml of CH_2_Cl_2_ was added to the residue and extracted three times with 5% NaOH. The organic layer was dried under sodium sulfate, filtered, and evaporated. The resulting product was used without further purification (yield 65%).

### Synthesis of 1-(4-(3-piperidin-1-yl)propoxy)phenyl)ethan-1-one (II) (CAS256952-65–5)

1-(4-(3-Bromopropoxy)phenyl)ethan-1-one (**I**) (0.6 mol; 11.49 g), piperidine (0.6 mol; 4.40 ml), potassium carbonate (0.75 mol; 10.39 g) and catalytic amount of potassium iodide were refluxed for 24 h in acetonitrile. After the solid was filtered and the solvent evaporated, the residue was purified by flash chromatography (CH_2_Cl_2_) to produce an orange oil (38%).

### General procedure for the synthesis of final compounds IIIa–IIIc

To 1-(4-(3-piperidin-1-yl)propoxy)phenyl)ethan-1-one (**II**) (1 mmol, 0.262 g) in 1 ml of absolute ethanol was added an appropriate aldehyde (1 mmol) and stirred for 5 min at room temperature. Then, while stirring, 0.5 ml of a 15% potassium hydroxide solution was slowly added dropwise and stirred for 30 min to 4 h at room temperature. The reaction was monitored by TLC. The TLC plates were stained with Dragendorff's reagent. Then, 2–3 ml of ice-cold water was added and a solution was acidified with concentrated hydrochloric acid (a few drops). The resulting precipitate was filtered, weighed, and then purified by crystallization from ethanol.

### (*E*)-3-(4-Methoxyphenyl)-1-(4-(3-(piperidin-1-yl)propoxy)phenyl)prop-2-en-1-one hydrogen chloride (IIIa)

Synthesis from 4-methoxybenzaldehyde (1 mmol; 0.12 mL). Yield 12% (49 mg), m.p. 174–177 °C, C_24_H_29_NO_3_ x HCl (MW = 415.96). ^1^H NMR (300 MHz, DMSO-d_6_) δ: 10.33 (br. s., 1H), 8.14 (d, *J* = 8.8 Hz, 2H), 8.03—7.41 (m, 4H), 7.22—6.58 (m, 4H), 4.16 (br s, 2H), 3.81 (s, 3H), 3.66 -3.32 (m, 2H), 3.15 (br s, 2H), 3.00—2.63 (m, 2H), 2.20 (br. s., 2H), 1.88–1,29 (m, 6H). LC/MS purity: 98.61%, *t*_*R*_ = 5.02 s, (ESI) m/z [M + H]^+^ 380.07.

### (*E*)-3-(3,4-Dimethoxyphenyl)-1-(4-(3-(piperidin-1-yl)propoxy)phenyl)prop-2-en-1-one hydrogen chloride (IIIb)

Synthesis from 3,4-dimethoxybenzaldehyde (1 mmol; 0.166 g). Yield 25% (110 mg), m.p. 173–176 °C, C_25_H_31_NO_4_ x HCl (MW = 445.98). ^1^H NMR (500 MHz, DMSO-d_6_) δ: 10.46 (br. s., 1H), 8.14 (d, *J* = 8.88 Hz, 2H), 7.80 (d, *J* = 15.47 Hz, 1H), 7.63 (d, *J* = 15.47 Hz, 1H), 7.50 (d, *J* = 1.72 Hz, 1H), 7.34 (dd, *J* = 1.72, 8.31 Hz, 1H), 7.05 (d, *J* = 8.88 Hz, 2H), 6.98 (d, *J* = 8.59 Hz, 1H), 4.15 (t, *J* = 6.01 Hz, 2H), 3.82 (s, 3H), 3.78 (s, 3H), 3.41 (d, *J* = 11.74 Hz, 2H), 3.09—3.19 (m, 2H), 2.76—2.89 (m, 2H), 2.14—2.26 (m, 2H), 1.70—1.83 (m, 4H), 1.67 (d, *J* = 13.17 Hz, 1H), 1.35 (dd, *J* = 6.73, 11.60 Hz, 1H). ^13^C NMR (126 MHz, DMSO-d_6_) δ: 187.7, 162.6, 151.7, 149.5, 144.3, 131.4, 128.1, 124.4, 120.0, 114.9, 112.0, 111.2, 66.0, 56.3, 56.1, 53.8, 52.5, 23.7, 22.9, 21.9. LC/MS purity: 100%, *t*_*R*_ = 4.71 s, (ESI) m/z [M + H]^+^ 410.30.

### (*E*)-3-(3,4,5-Trimethoxyphenyl)-1-(4-(3-(piperidin-1-yl)propoxy)phenyl)prop-2-en-1-one hydrogen chloride (IIIc)

Synthesis from 3,4,5-trimethoxybenzaldehyde (1 mmol; 0.196 g). Yield 20% (95 mg), m.p. 176 °C dec., C_26_H_33_NO_5_ x HCl (MW = 476.00). ^1^H NMR (500 MHz, DMSO-d_6_) δ: 10.48 (br s, 1H), 8.16 (d, *J* = 8.88 Hz, 2H), 7.87 (d, *J* = 15.47 Hz, 1H), 7.63 (d, *J* = 15.47 Hz, 1H), 7.19 (s, 2H), 7.06 (d, *J* = 8.88 Hz, 2H), 4.15 (t, *J* = 5.87 Hz, 2H), 3.83 (s, 6H), 3.67 (s, 3H), 3.40 (d, *J* = 11.46 Hz, 2H), 3.13 (d, *J* = 5.16 Hz, 2H), 2.83 (d, *J* = 11.46 Hz, 2H), 2.21 (qu, *J* = 6.01 Hz, 2H), 1.70—1.87 (m, 4H), 1.66 (d, *J* = 13.17 Hz, 1H), 1.35 (br. s., 1H). ^13^C NMR (126 MHz, DMSO-d_6_) δ: 187.8, 162.7, 153.6, 144.3, 140.1, 131.5, 131.2, 130.9, 121.7, 115.0, 107.0, 66.0, 60.7, 56.7, 53.8, 52.5, 23.7, 22.8, 21.9. LC/MS purity: 100%, *t*_*R*_ = 4.84 s, (ESI) m/z [M + H]^+^ 440.34.

### Histamine H_3_ receptor affinity

Radioligand binding studies were conducted using CHO-K1 cells stably transfected with the human H_3_ receptor as we described previously [38]. All assays were carried out in duplicates. [^3^H]-*N*-α-methylhistamine was used as radioligand and (R)(-)-α-methylhistamine was used to define nonspecific binding. Radioactivity was counted in MicroBeta2 scintillation counter (PerkinElmer). Data were fitted to a one-site curve-fitting equation with Prism 6 (GraphPad Software) and *K*_*i*_ values were estimated from the Cheng − Prusoff equation.

### Histamine H_1_ and H_4_ receptor affinity

Radioligand binding studies, using CHO-K1 cells stably expressing the H_1_R or S*f*9 cells transiently expressing the H_4_R were performed as described previously [39]. [^3^H]Pyrilamine (H_1_R) and [^3^H]histamine (H_4_R) were used as radiolignads. Affinities were determined using at least five different concentrations of tested compounds in triplicate independent experiments.

### Cell line and culture conditions

The murine microglial BV-2 cell line was purchased from the Interlab Cell Line Collection (ICLC) cell bank (Genova, Italy). The BV-2 cells were cultured in DMEM/F12 medium (Lonza, BioWhittaker, Walkersville, MD USA, 12-719F) containing 10% of heat-inactivated fetal bovine serum (FBS, Gibco, 10,500–064) in a humidified incubator containing 95% air and 5% CO_2_ at 37 °C. BV-2 cells were used for experiments at passages 3 to 11. In all experiments, the concentration of FBS was reduced to 5%.

### Cell viability assay

BV-2 cells (2 × 10^4^ cells/100 μl/well) were cultured in transparent 96-well plates (TPP, Trasadingen, Switzerland) in DMEM/F12 supplemented with 5% FBS in the presence of dimethylsulfoxide (DMSO ≤ 0.1%, vehicle control, Veh) or increasing doses of tested compounds (0.78 × 10^–6^ – 50 × 10^–6^ M). Compound treatment was performed for 24 h. After the incubation time, cell viability was examined using an MTS-based [3-(4,5-dimethylthiazol-2-yl)-5-(3-carboxymethoxyphenyl)-2-(4-sulfophenyl)-2 H tetrazolium] CellTiter96® AQueous One Solution Cell Proliferation Assay (Promega, Madison, USA) following the manufacturer’s protocol. Briefly, 20 μl of MTS solution was pipetted into each well containing 100 μl of culture or culture medium without cells (blank control), mixed well and incubated at 37 °C for 55 min. After the appropriate time, the absorbance was measured at 490 nm using the EnSpire microplate reader (PerkinElmer, Massachusetts, USA). IC_50_ values were calculated with non-linear regression fit to a sigmoidal dose–response curve (log of compound concentration versus normalized response) using GraphPad Prism (v. 4.0.3).

### Nitrite oxide (NO) assay (Griess assay)

The Griess assay is a colorimetric assay to measure the levels of nitrite (NO_2_^−^) which is one of the primary and stable metabolites of NO. Griess reagent (Sigma-Aldrich, Saint Louis, MO, USA, G4410) was freshly prepared according to the manufacturer's instructions, that is, 1 g was dissolved in ultrapure water and mixed by inversion for about 4 min. For measuring nitric oxide, after an appropriate incubation time, 100 μl of culture medium was transferred to a transparent 96-well plate and mixed with the same volume of Griess reagent. The plate was incubated for 15 min at room temperature and the absorbance was read at 540 nm using the EnSpire microplate reader (PerkinElmer, Massachusetts, USA). Data were analysed by direct comparison based on the average absorbance of LPS treatment, compound treatment or co-treatment with LPS, performed by normalizing treated cells (compound alone or co-treatment with LPS) to LPS-treated cells set as 100%.

### Total RNA isolation and real-time qPCR

For the real-time qPCR (RT-qPCR) experiment, BV-2 cells were seeded on a 24-well plate (VWR, Matsonford Road, Radnor, PA, USA, 734–2325) at a density of 15 × 10^4^ cells/500 μl/well in DMEM/F12 supplemented with 10% FBS. The next day, the culture medium was replaced with treatment medium (5% FBS) according to the following scheme: 1) DMSO (0.1%; Veh); 2) LPS (1 μg/ml); 3) tested compounds (6.25 μM) 4) 1 h pretreatment with tested compounds followed by LPS 1 μg/ml. The experiment was carried out in triplicate and at least three independent experiments were carried out. Total RNA was extracted from BV-2 cells with TRIzol® reagent (Invitrogen, Carlsbad, CA, USA), based on the guanidine thiocyanate method described by Chomczynski and Sacchi with some modification [40]. Briefly, at the end of the treatment, the culture medium was collected, centrifugated, transferred to a new Eppendorf tube and kept at  – 80 °C for further ELISA analysis. The remaining cells were lysed on a plate at 4 °C for 10 min by adding 500 μl of TRIzol® reagent to each well. After this time, the lysate was pipetted several times for better homogenization and transferred to a new Eppendorf tube. Next, 150 μl of chloroform was added, the samples were shaken vigorously (not vortex) and kept on ice for 15 min followed by centrifugation for 20 min at 18,000 rpm and 4 °C (Mikro 22R, Hettich, Tuttlingen, Germany). The aqueous phase was carefully transferred to a new Eppendorf tube and 250 μl of isopropanol was added followed by chilling for 20 min at  – 20 °C. Subsequently, the tubes were centrifuged for 30 min at 5000 rpm and 4 °C. Total RNA precipitates were washed three times with cold 70% ethanol, including centrifugation between each wash for 10 min at 15,000 rpm. The RNA pellet was air dried and dissolved in 20 μl of DEPC-treated water (IBI Scientific, USA). Total RNA quantification was performed spectrophotometrically (Eppendorf BioPhotometer, Eppendorf Scientific, Inc., Westbury, NY) in a 50 μl microcuvette (1 μl of RNA sample + 49 μl of DEPC-treated water). Only the RNA samples with absorbance ratio at A260/A280 = 1.8–2.2 and A260/A230 = 1.5–2.0 were used for further analysis. Reverse transcription was performed on 500 ng of total RNA using High Capacity cDNA Reverse Transcription Kits (Applied Biosystem, Foster City, CA) and Mastercycler gradient thermal cycler (Eppendorf, Germany), following the manufacturer’s instruction. The cDNA products were then diluted with nuclease-free water in a 1:10 ratio and used in RT-qPCR analysis. The expression of target genes and *HPRT1* as a housekeeping gene was performed using TaqMan Gene Expression Assays (summarized in the Table 1) and StepOnePlus™ real-time PCR system (Applied Biosystems) according to the manufacturer’s recommendations. The threshold levels (*C*_*t*_ value) for each gene were automatically derived and calculated by StepOnePlus™ software. The abundance of RNA was calculated as 2^−ΔΔCt^. The expression of the housekeeping gene (*HPRT1*) was quantified to control for variation in cDNA amounts.

**Table 1:** List of RT-qPCR genes and assay IDs

Gene	Assay ID
HPRT1	Mm00446968_m1
IL-1β	Mm00434228_m1
IL-6	Mm00446190_m1
TNF-α	Mm00443258_m1

### Enzyme-linked immunosorbent assay

The concentrations of IL-1β, IL-6, TNF-α, the main inflammatory cytokines, were analysed using commercial ELISA kits (IL-6 ELISA kit, ADI-900–045, TNF-α ELISA kit, ADI-900–047, Enzo, Mouse IL-1 beta ELISA kit, Invitrogen) according to the manufacturer’s instructions. Briefly, at the end of 24 h of incubation with tested compounds, cell culture plates were centrifuged to remove cell debris from the supernatants. Culture medium was collected in Eppendorf tubes and frozen at −80 °C until further analysis.

### In vitro neuroinflammation model of LPS-treated BV-2 cells

To establish an experimental model of neuroinflammation in vitro*,* BV-2 cells were cultured in a 96-well plate (2 × 10^4^ cells/200 μl/per well) and a 24-well plate (15 × 10^4^ cells/500 μl/well) for the MTS assay and the Griess assay or the RT-qPCR experiment, respectively. BV-2 cells were treated for 24 h, 45 h and 69 h with the increasing concentration of LPS (Sigma-Aldrich, Saint Louis, MO, USA, L4391): 0.001; 0.01; 0.1; 1 [μg/ml]. Following LPS stimulation, at each time point, 100 μl of culture medium from each sample was transferred to another plate and mixed with an equal volume of Griess reagent. Simultaneously, on the plate with cells, cell viability was assessed by adding 20 μl of MTS reagent. The cells cultured on a 24-well plate were used for assessing the expression levels of proinflammatory cytokines (IL-1β, IL-6, TNF-α) by RT-qPCR.

### Immunocytochemistry

BV-2 cells were cultured using the Nunc™ Lab-Tek™ II 8-well Chamber Slide™ System (Thermo Fisher Scientific), 4 × 10^4^ cells were seeded into each well and the following day were inoculated with 1 µg/ml LPS and stimulated for a further 24 h. After that, cells were fixed with 4% PFA for 15 min at room temperature and subsequently washed with PBS. Samples were prepared as for classical IHC staining described above, heated (95 °C for antigen retrieval in 5% urea in 100 mM TRIS (pH = 9.5) solution, permeabilized with PBS contained 0.25% Triton-X-100, blocked in 5% normal donkey serum (017–000-121, Jackson Immnuresearch) in PBS and incubated overnight in 4 °C with proper primary antibodies diluted in 5% NDS or SignalBoost™ Immunoreaction Enhancer Kit (407,207, Merck Millipore, USA): anti-IBA1 (1:500, 019–19,741; Wako, Japan), anti-B7-2/CD86 (1:500; AF1340, R&D Systems, USA), anti-CD206 (1:500; sc-58986, Santa Cruz Biotechnology, USA). After three washes in PBS, triple immunofluorescence was revealed by incubation for 2 h in the appropriate fluorochrome-conjugated secondary antibody: donkey anti-mouse Alexa Fluor488 (A-21202, Invitrogen), donkey anti-rabbit Alexa Fluor594 (A-21207, Invitrogen) donkey anti-goat Alexa Fluor647 (705-605-003, Jackson ImmunoResearch) diluted 1∶500 in 5% NDS. Sections were then washed and coverslipped with an Aquatex mounting medium (Merck, Darmstadt, Germany). Leica TCS SP8 WLL confocal microscope was used to take the pictures. Images were processed in Leica LAS X microscope software.

### Statistical analysis

All statistical analyses were carried out using GraphPad Prism 7 with significance determined by One-way ANOVA followed by post-hoc comparisons tests as detailed in the figure legends.

## Results

### Histamine H_3_ receptor activity of tested compounds

Compounds **IIIa**–**IIIc** were tested for affinity for human H_3_R (*h*H_3_R) in a radioligand binding assay with *N*^α^-methylhistamine as a radioligand in CHO K1 cells stably expressing *h*H_3_R. Results are presented as *K*_*i*_ values with SEM (Table 2). In general, all compounds showed very good affinities with *K*_*i*_ values < 25 nM. The most potent was compound **IIIb** with a dimethoxy substituent in the phenyl ring (*K*_*i*_ = 9.6 nM). The affinity of this compound was comparable to a derivative with one methoxy group in the phenyl ring, compound **IIIa** (*K*_*i*_ = 14 nM). It appears that the introduction of the third methoxy group into the 5 position in the phenyl ring (**IIIc**) slightly decreased the affinity of *h*H_3_R (*K*_*i*_ = 24 nM). However, it is still a very active compound. The affinity for *h*H_3_R (stably expressed in HEK293 cells) of compound **E243** was recently described [37]. This compound is a ligand with moderate affinity for *h*H_3_R (*K*_*i*_ = 228.2 nM).

**Table 2:**
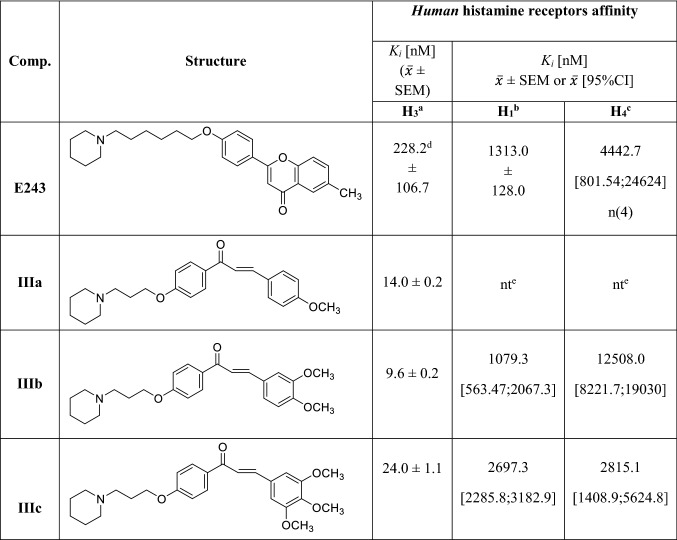
Pharmacological activity of compounds **E243** and **IIIa**-**IIIc**

^a^[^3^H] *N*^α^-Methylhistamine binding assay performed with cell membrane preparation of CHO-K1 cells stably expressing the human H_3_ receptor; mean value of two independent experiments ± SEM

^b^[^3^H]Pyrilamine binding assay performed with cell membrane preparation of CHO-K1 cells stably expressing the human histamine H_1_ receptor; mean value of at least triplicate independent experiments ± SEM or within the 95% confidence interval (CI)

^c^[^3^H]Histamine binding assay performed with cell membrane preparation of S*f*9 cells transiently expressing the human histamine H_4_ receptor, co-expressed with G protein Gα_i2_ and Gß_1γ2_ subunits; mean value of at least triplicate independent experiments within the 95% confidence interval (CI)

^d^[^3^H] *N*^α^-Methylhistamine binding assay performed with cell membrane preparation of HEK293 cells stably expressing the human H_3_ receptor; mean value of triplicate independent experiments; data from Bajda et al*.* [35]

^e^*nt* not tested

### Histamine H_1_ and H_4_ receptor activity of E243, IIIb and IIIc

Selected compounds were further evaluated for their binding affinity at related histamine receptor subtypes: human histamine H_1_ receptor (*h*H_1_R) and human H_4_ receptor (*h*H_4_R). Results are collected in Table 2. All tested compounds showed micromolar affinity at *h*H_1_R and *h*H_4_R. A selectivity for *h*H_3_R over these receptors was good (> 100) for chalcones **IIIb** and **IIIc** (**IIIb**: *K*_*i*_ (H_4_R)/*K*_*i*_ (H_3_R) > 1300, *K*_*i*_ (H_1_R)/*K*_*i*_ (H_3_R) > 112; **IIIc**: *K*_*i*_ (H_4_R)/*K*_*i*_ (H_3_R) > 117, *K*_*i*_ (H_1_R)/*K*_*i*_ (H_3_R) > 112). However, compound **E243** proofed to be non-selective, especially towards H_1_R: *K*_*i*_ (H_4_R)/*K*_*i*_ (H_3_R) > 19 and *K*_*i*_ (H_1_R)/*K*_*i*_ (H_3_R) > 5.

### Optimization of LPS concentration for functional in vitro neuroinflammation model

**Fig. 2:**
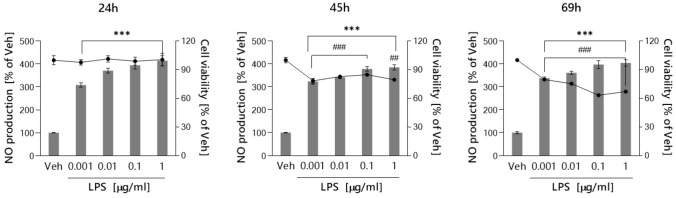
Optimization of LPS concentration based on Griess (NO level, bar graph) and MTS assay (cell viability, line graph). Each point represents the mean ± SEM of three independent experiments, each of which consisted of three replicates per treatment group. Data are presented as a percentage of control cells treated with 0.1% DMSO (Veh). Statistical analysis by one-way ANOVA with post-hoc Dunnett’s test at significance level α = 0.05 (***p < 0.001, MTS assay; ###*p* < 0.001, ##*p* < 0.01, Griess assay)

**Fig. 3:**
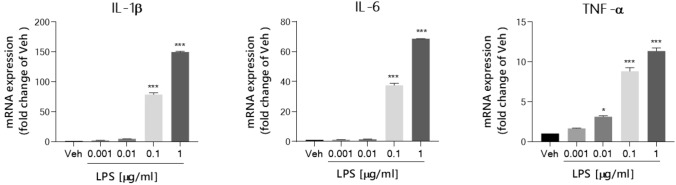
Optimization of LPS concentration based on the expression of pro-inflammatory cytokines. Data is present as relative changes of mRNA expression of *IL-1β*, *IL-6* and *TNF-α* analysed by quantitative real-time PCR. Each point represents the mean ± SEM of three independent experiments, each of which consisted of three replicates per treatment group. Data are presented as a percentage of control cells treated with 0.1% DMSO (Veh). Statistical analysis by one-way ANOVA with post-hoc Tukey’s test at significance level α = 0.05 (*p < 0.1, ***p < 0.001)

Initially, our objective was to establish a functional in vitro model of neuroinflammation in murine microglial BV-2 cells. For this, we first examined the effect of increasing concentration of LPS (0.001 – 1 μg/ml) on the viability of BV-2 cells at three time points: 24 h, 45 h, and 69 h. The goal of this experiment was to select an appropriate dose of LPS that would induce an increase in nitric oxide (NO)—a commonly assessed neuroinflammatory endpoint—without causing a cytotoxic effect on cells. Our experimental results showed that LPS in all concentration ranges tested significantly induced NO level after 24 h of treatment without causing any toxic effect on BV-2 cells (Fig. 2). The highest increase, fourfold compared to control cells, was observed at an LPS concentration of 1 μg/ml. Although LPS at all concentrations tested induced NO production at a similar level at longer incubation times (45 h and 69 h), it reduced cell viability by an average of 22% and 37%, respectively. To better optimize LPS concentration, pro-inflammatory cytokine expression levels were also examined. As shown in Fig. 3, IL-1β, IL-6, and TNF-α mRNA expression of *IL-1β*, *IL-6*, and *TNF-α* was dose-dependent in response to LPS range concentration. Therefore, the results demonstrated that the optimal concentration of LPS and the duration of exposure for the next experiment were 1 μg/ml and 24 h, respectively.

**Fig. 4:**
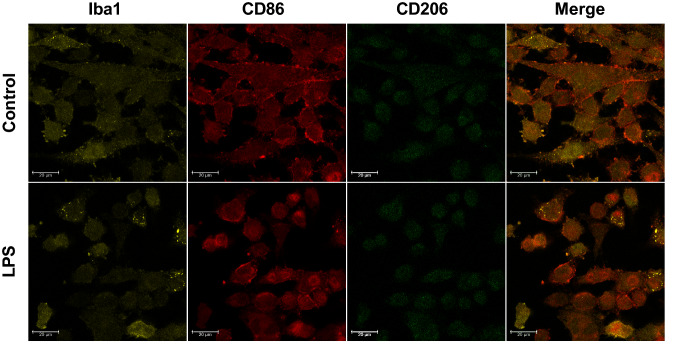
The presence of microglia markers (Iba1, CD86 and CD206) in control and LPS-stimulated BV-2 cells. Using immunocytochemistry we found that Iba1 (orange), CD86 (red) and CD206 (green) are expressed in BV-2, both in control (upper panel) and LPS-treated (lower panel) cells. The scale bar for all microphotographs is 20 µm

Our immunocytochemistry studies revealed an expression of microglial cell markers (Iba1, CD86, CD206) in both control and LPS-stimulated BV-2 cells (Fig. 4). Moreover, most BV-2 cells showed a round shape after LPS treatment, which is one of the sings of microglia activation.

### Effect of compounds E243 and IIIa – IIIc on the viability of BV-2 cells

**Fig. 5:**
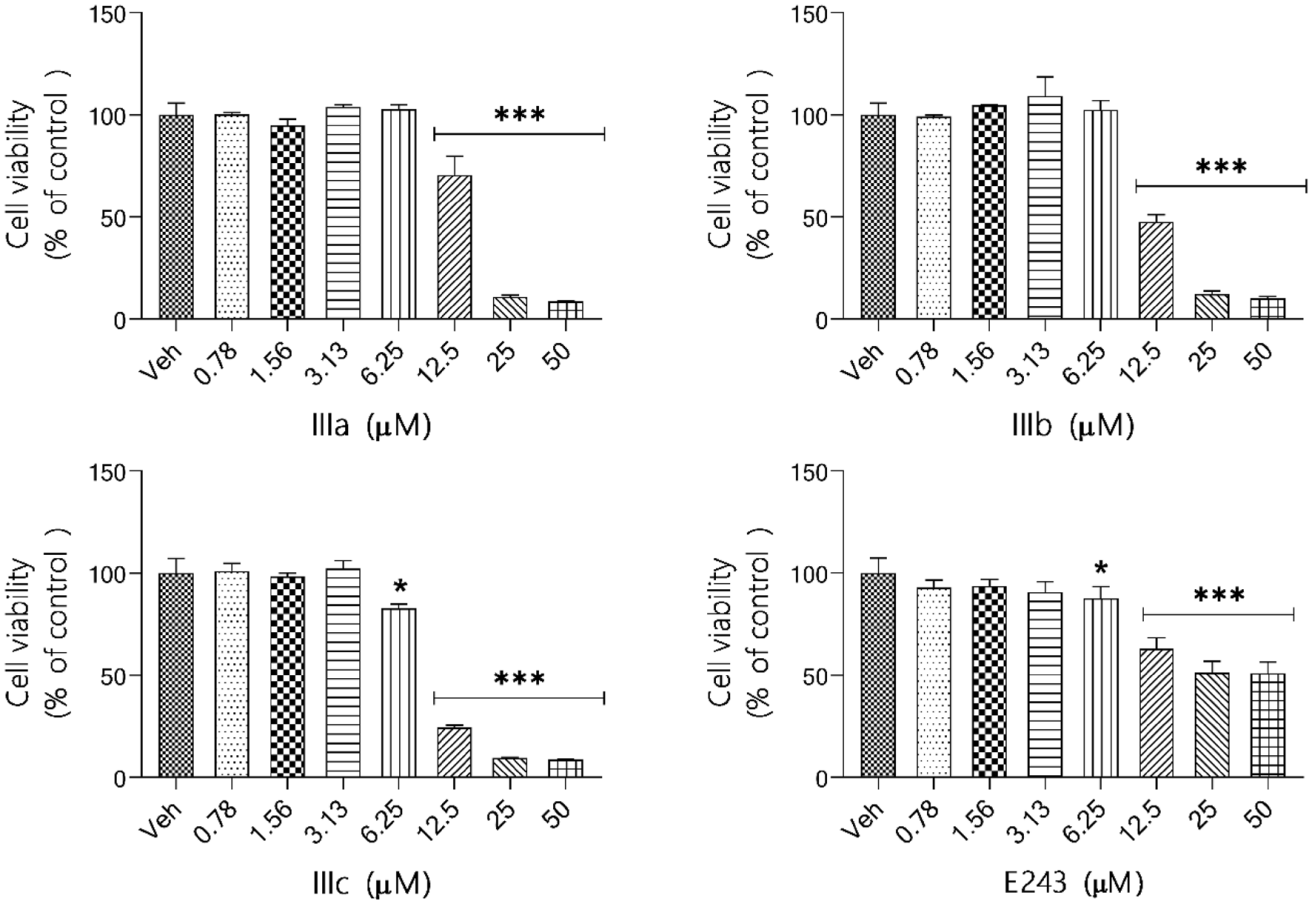
Effect of compounds **E-243**, **IIIa–IIIc** on BV-2 cell viability. BV-2 cells were incubated for 24 h in the presence of increasing concentrations of the compounds tested (0.78 × 10^–6^ – 50 × 10^–6^ M). Cell viability was measured by an MTS assay. Each point represents the mean ± SEM of three independent experiments, each of which consisted of three replicates per treatment group. Data are presented as a percentage of control cells treated with 0.1% DMSO (Veh). Data were analyzed by one-way ANOVA followed by the Dunnett’s multiple comparison test (*α* $=$ 0.05). Data indicated with *p $<$ 0.05; ***p $<$ 0.001 (versus vehicle-treated cells) reflect statistically significant differences between the control and experimental groups

To avoid the toxic effect of H_3_R ligands in subsequent studies, an MTS assay was performed to investigate cell viability. BV-2 cells were cultured for 24 h in the presence of an increasing concentration of compounds **E243** and **IIIa–IIIc** (twofold dilutions covered the range from 0.78 × 10^–6^ to 50 × 10^–6^ M). A dose–response effect is presented on Fig. 5 and an IC_50_ values are summarized in Table 3. All compounds tested at a concentration of 6.25 μM had no effect on BV-2 cell viability and were chosen for further anti-inflammatory studies.

**Table 3:** IC_50_ values determined by fitting a sigmoidal dose–response curve to the data using Graph Pad Prism

Compound	IC_50_^a^ $\overline{x }$ ± SD (μM)
E243	53.46 ± 5.56
IIIa	21.19 ± 1.98
IIIb	26.69 ± 3.48
IIIc	11.34 ± 3.26

^a^The mean value of IC_50_ from the MTS assay in BV-2 cells at the 24 h of exposure. The IC_50_ value of each compound was defined as the concentration (μM) that caused 50% inhibition of cell viability in BV-2 cells compared to vehicle-treated cells

### Preliminary evaluation of the anti-inflammatory activity of compounds E243 and IIIa – IIIc in LPS-stimulated BV-2 cells

**Fig. 6:**
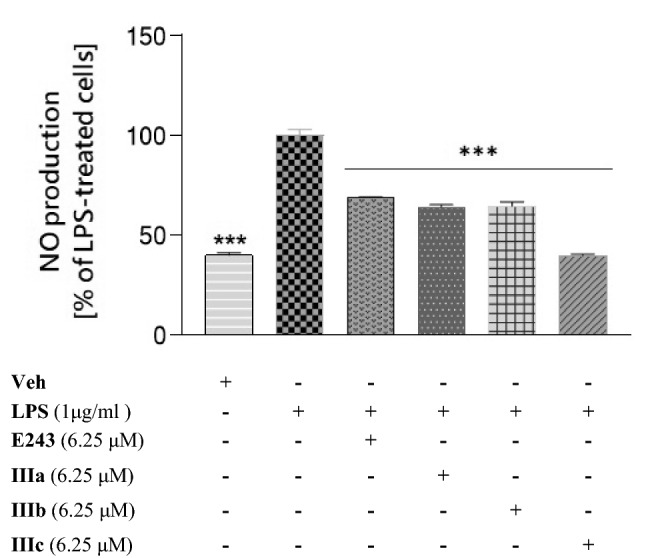
The effect of the compounds tested (**E243** and **IIIa–IIIc**) on nitric oxide (NO) production in BV-2 cells assessed by the Griess assay. BV-2 cells were pretreated for 1 h with indicated compounds at the concentration of 6.25 μM, followed by stimulation with 1 μg/ml LPS for 24 h. Data are expressed as mean ± SEM of two independent experiments, each of which consisted of eight replicates per treatment group and is expressed as a percentage of NO production versus cells treated with LPS (set at 100%). Statistical analysis by one-way ANOVA showed significant differences between the groups (α $=$ 0.05) and was followed by the Dunnett’s multiple comparison test. Data indicated with ***p $<$ 0.001 (versus vehicle-treated cells) reflect statistically significant differences between the control and experimental groups

To evaluate the anti-inflammatory effects of H_3_R ligands on LPS-stimulated BV-2 cells, the level of NO in cell culture medium was measured by the Griess reagent assay. As shown in Fig. 6, the level of NO decreased significantly after LPS stimulation in the presence of compounds **E243** and **IIIa**–**IIIc**. Compounds **E243**, **IIIa**, and **IIIb** reduced the level of NO by an average of 30% compared to LPS treated cells, while **IIIc** reduced this level by 61%. Because compound **IIIc** reduced NO concentration twice more than the other tested compounds, it was selected for further study.

### Compound IIIc suppressed LPS-induced mRNA expression of pro-inflammatory cytokines in BV-2 cells

**Fig. 7:**
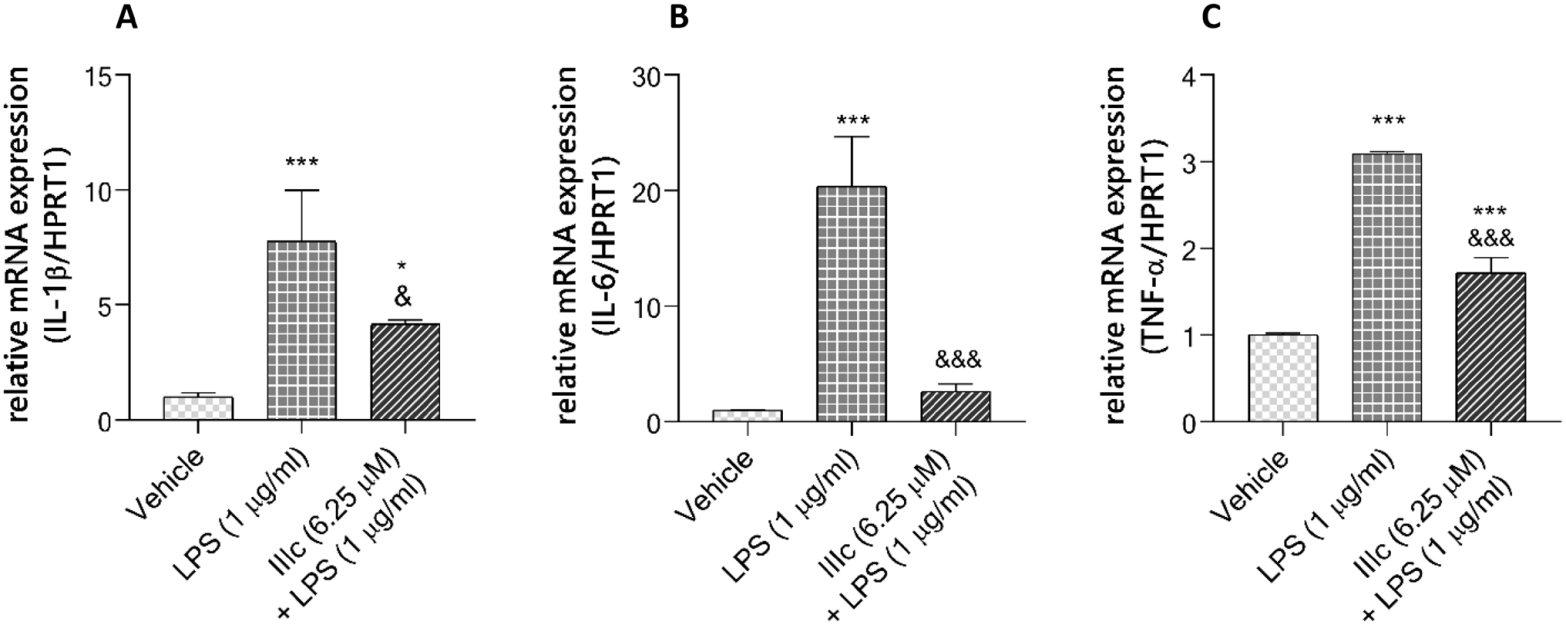
Effect of compound **IIIc** on gene expression of pro-inflammatory cytokines in LPS-activated BV-2 cells. Cells were pretreated with 6.25 μM **IIIc** 1 h prior to incubation with LPS (1 μg/ml) for 24 h. mRNA *IL-1β* (**A**), *IL-6* (**B**) and *TNF-α* (**C**) were determined by quantitative real-time PCR as described in Material and Methods. The relative level of mRNA was normalized to the expression of HPRT mRNA. The results are expressed as mean ± SD for each group from three independent experiments. Statistical analysis by one-way ANOVA showed significant differences between the groups (*α* $=$ 0.05) and was followed by Fisher’s LSD test. Data indicated with ***p $<$ 0.001, *p $<$ 0.05 (versus vehicle-treated cells) and &&&*p* $<$ 0.001, &*p* $<$ 0.05 (versus LPS-treated cells) reflects statistically significant differences between control and experimental groups

To investigate the effect of **IIIc** on the expression of pro-inflammatory cytokines, BV-2 cells were treated with LPS in the presence or absence of the compound tested for 24 h. Figure 7 shows a clear trend of decreasing the release of pro-inflammatory cytokines after **IIIc** treatment, indicating that this compound has anti-inflammatory activity. We found that *IL-6 *mRNA levels after cotreatment with LPS and **IIIc** were 10 times lower compared to those induced by LPS alone and that was the most significant decline. mRNA levels of *IL-1β* and *TNF-α* were on average 2 times and 3 times lower, respectively, than those induced by LPS alone.

### Compound IIIc represses LPS-induced production of pro-inflammatory cytokines in BV-2 cells

**Fig. 8:**
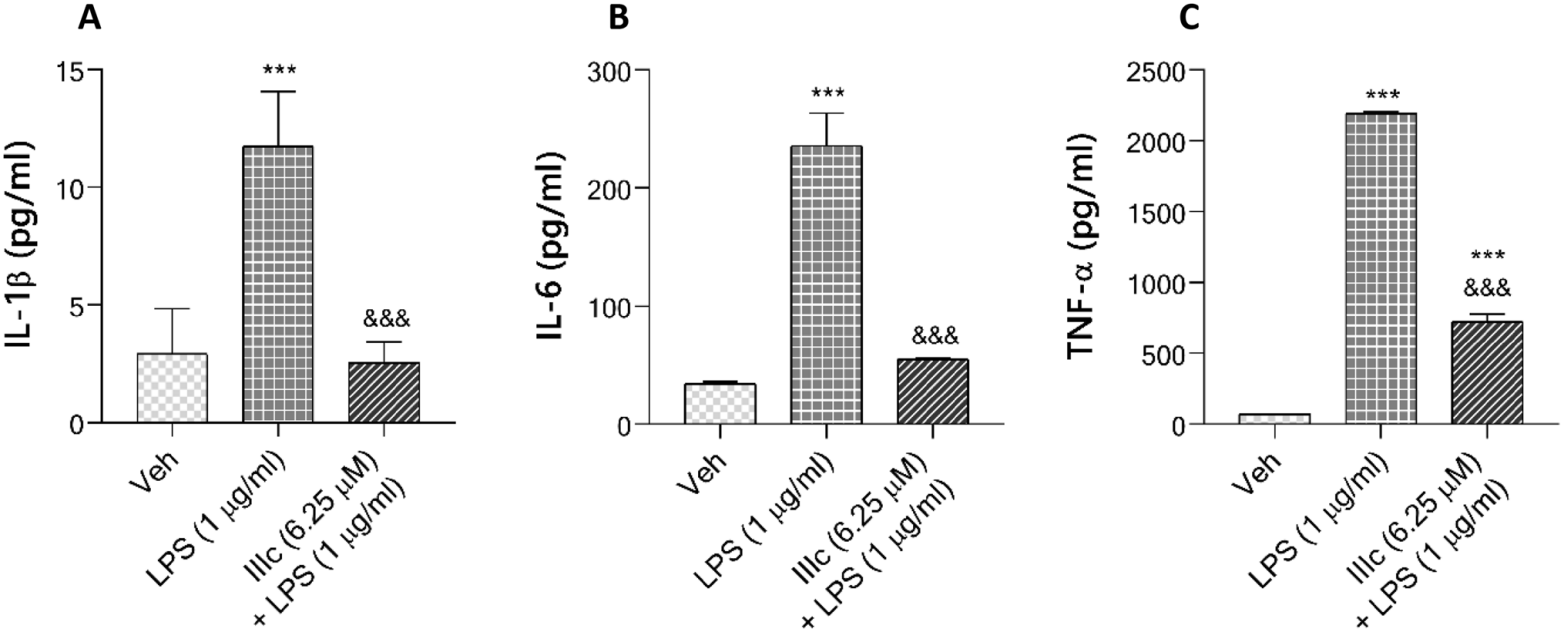
Effect of compound **IIIc** on the production of proinflammatory cytokines by LPS-activated BV-2 cells. Cells were pretreated with 6.25 μM **IIIc** 1 h prior to incubation of LPS (1 μg/ml) for 24 h. The release of IL-1β (**A**), IL-6 (**B**) and TNF-α (**C**) in the cell culture supernatant were analysed using ELISA as described in Material and Methods. Results are expressed as mean ± SD for each group from three independent experiments. Statistical analysis by one-way ANOVA showed significant differences between the groups (α $=$ 0.05) and was followed by Fisher’s LSD test. Data indicated with ***p $<$ 0.001, (versus vehicle-treated cells) and &&&*p* $<$ 0.001 (versus LPS-treated cells) reflects statistically significant differences between control and experimental groups

IL-1β, IL-6 and TNF-α are critical pro-inflammatory cytokines in response to LPS. Thus, we decided to check whether **IIIc** affected their production using ELISA. The results of this test are presented in Fig. 8. Co-treatment with **IIIc** in the presence of LPS prevents the induction of pro-inflammatory cytokines. The release of IL-1β, IL-6 and TNF-α were significantly lower compared to LPS-stimulated BV-2 cells. These results showed that protein expression results correlate with mRNA expression results, further proving the anti-inflammatory nature of the compound tested.

## Discussion

Our initial studies aimed to examine the effect of H_3_R antagonists on expression and secretion of pro-inflammatory cytokines in murine microglia after LPS stimulation. LPS is a widely used stimuli to model neuroinflammation associated with neurodegeneration [41]. From the vast amount of literature on neurodegenerative diseases such as AD, PD or amyotrophic lateral sclerosis, it appears that they involve some pathological factor that negatively influences the elements of the CNS, such as structure and survival of neuronal cells. In effect, damaged neurons provide a clear inflammatory stimuli to local microglia—the first and main form of active innate immune defence in the CNS. In such condition, microglia are activated which is associated with the production and secretion of variety of mediators such as pro-inflammatory cytokines and NO. However, uncontrolled inflammatory responses can enhance oxidative stress and trigger apoptotic cascades in neurons [42]. Thus, chronic neuroinflammation is an undesirable phenomenon that plays an important role in the development and progression of neurodegenerative diseases [43, 44]. For this reason, it is considered highly likely that a modulation of microglia could be a therapeutic target in neurodegenerative diseases.

The main challenge for this study was to evaluate the anti-inflammatory efficacy of H_3_R antagonists in a functional in vitro model of neuroinflammation. For this purpose, we first optimized the concentration of LPS that is adequate to significantly induced the signs of neuroinflammation. It was shown that 1 μg/ml of LPS stimulated BV-2 cells to remarkably produce the level of NO and expression of pro-inflammatory cytokines such as IL-1β, IL-6 and TNF-α. These results are in line with those obtained by Hosseini et al*.* who strictly focused on optimization the conditions of inflammation in BV-2 cell [45]. The above concentration is also commonly used in experimental work by other researchers [46, 47]. Here, we also revealed that BV-2 cell line express markers typical for microglia (for review see [48]) such as Iba1, CD68 and CD206. Moreover, after LPS stimulation, BV-2 took a round shape, which indicates the activation of these cells. Our data has strongly suggested that BV-2 might by a useful tool for study microglia physiology.

Next we checked whether compound **E243** (a substituted flavone), and compounds **IIIa-IIIc** (substituted chalcones) could reduce NO production in LPS-stimulated BV-2 cells. Results indicated that all tested compounds reduced the level of NO, but only one of the chalcones (**IIIc**) showed the most potent inhibitory activity and lowered this level by as much as 61% compared to control cells (not treated with LPS). The most interesting for us at this stage of investigation was to establish whether this data correspond to the lower level of pro-inflammatory cytokines. The analysis of genes expression was evaluated by real-time qPCR. We found that the IL-6 mRNA level was ten times lower expressed when BV-2 cells were co-treated with **IIIc** instead of LPS alone. Satisfactory results were also obtained for mRNA levels of *IL-1β* and *TNF-α*. It is worth noting that, we also have shown that the results of mRNA expression correlate with that of protein expression evaluated by ELISA. These findings further strongly support our hypothesis that H_3_R antagonist based on the chalcone scaffold could be valuable in inflammation research.

The anti-neuroinflammatory potential of chalcones has also been documented in other in vitro and in vivo studies [49]*.* Yang et al*.* demonstrated that Safflower yellow, which is a mixture of water soluble chalcone, significantly inhibited the production of IL-1β, IL-6, TNF-α in LPS-stimulated BV2 cells [50]. Moreover, in the study presented by Mateeva et al*.*, it was shown that 2′-hydroxy-3,4,5,3′,4′-pentamethoxychalcone was shown to represent a basic structure with the most potent anti-inflammatory effect, evidenced by complete inhibition of NO and the reduction of various pro-inflammatory cytokines in activated BV-2 cells [51]. This finding broadly supports the work of other groups of researchers in this area linking the number of methoxy groups in the phenyl ring with the capacity to suppress the overactivated immune response of microglial cells [52]. Furthermore, Zhang et al. identified methoxy chalcones as a potential candidate for the treatment of acute inflammatory diseases [53]. This is even more interesting as all methoxy chalcones examined in our study showed very good affinities for *h*H_3_R with K_i_ values < 25 nM. However, the most potent compound **IIIb** with a dimethoxy substituent on the phenyl ring (*K*_*i*_ = 9.6 nM) and its derivative with one methoxy group on the phenyl ring, compound **IIIa** (*K*_*i*_ = 14 nM) have not shown inhibitory activity on pro-inflammatory cytokine production in LPS-stimulated BV-2 cells (data not shown). It appears that the introduction of the third methoxy group into the 5 position of the phenyl ring (**IIIc**) greatly improved anti-inflammatory properties even though it slightly decreased the affinity for *h*H_3_R (*K*_*i*_ = 24 nM). However, it is still a very active compound.

Contrary to expectations, this study did not confirmed the anti-inflammatory properties of compound **E243**. It is somewhat surprising as our previous research described **E243** as a multi-target compound against AD [37]. We stated that one of the interesting pharmacological approaches is to use a single molecule that modulates multiple targets simultaneously. The combination of acetyl- (AChE) and butyrylcholinesterase (BuChE) inhibition with additional properties, e.g., H_3_R antagonism could be beneficial for slowing AD progression [54]. In this context, **E243** is very interesting as displayed submicromolar activity toward all aforementioned targets (human H_3_R *K*_*i*_ = 228 nM; human AChE IC_50_ = 360 nM; electric eel AChE IC_50_ = 501 nM; equine BuChE IC_50_ = 758 nM). Additionally, we examined the neuroprotective efficacy and showed that pre-treatment of neuroblastoma SH-SY5Y cells for 24 h with **E243** (at 1 µM concentration) significantly prevented ROS production (assessed by DCFH-DA assay) almost by 2 times compared to the cells treated with the following neurotoxins H_2_O_2_ (300 μM) and okadaic acid (50 nM) (presented at 2nd International Conference on Neuroprotection by Drugs, Nutraceuticals and Physical Activity, 9–10 Dec 2021, Italy). Compound **E243** has been proofed as neuroprotective agent to be effective in neuroblastoma cells. The fact that, **E243** did not act directly on microglia, might be due to the fact, that its activity is based on different mechanism not related to anti-inflammatory activity. Moreover, this can be partly explained by Qiao et al. who noticed that for anti-inflammatory activity of flavonoids the presence of free hydroxyl group(s) in the phenyl ring (ring B according to [33]) is important. With the increase of number of groups, the activity extent up to specific level. This observation can explain lack of anti-inflammatory activity of **E243**. This compound lacks any free hydroxyl group in the phenyl ring.
